# 

**Published:** 1831-01-01

**Authors:** 


### MEDICO-CHIRURGICAL

### REVIEW,

AND

### JOURNAL

OF

### PRACTICAL MEDICINE.

*(NEW SERIES.)*

VOLUME FOURTEEN,

[1st of OCTOBER, to 31st of MARCH,]

1831.

VOL. XVIII. of ANALYTICAL SERIES.

Nothing to index

EDITED BY JAMES JOHNSON, M.D.

PHYSICIAN EXTRAORDINARY TO THE KING,

&c. &c. &c.

Surgeon Gen'l's Office LIBRARY. 57453

LONDON:

PUBLISHED BY S. HIGHLEY, 174, FLEET STREET,

AND WEBB STREET, ST. THOMAS'S HOSPITAL,

And all other Booksellers.

WI
ME 75 T

PRINTED BY G. HAYDEN,

Little College Street, Westminster.

### BIBLIOGRAPHICAL RECORD;

1. An Address, introductory to a Course of Lectures on the Principles and Practice of Physic, delivered on Wednesday, 23d June, 1830, dedicated to the Members of the City of London Medical and Chirurgical Society. By James Baker, Surgeon.

2. De Tabulis Mortuorum, Vivorum, et Nascientium; necnon de Numero et Proportione diversorum Morborum, &c. A. Gulielmo Harty, M.B. Dublini, 1830.

☞ *A very ably executed thesis.*

---

3. Transactions of the Medical and Physical Society of Calcutta. Volume the Fourth. Octavo, pp. 445, with Plates, Calcutta, 1829.

---

4. A Demonstration of the Nerves of the Human Body, consisting of four Parts.—Part 1. The Cervical and Thoracic Portions of the Sympathetic, and the Nerves of the Thoracic Viscera—Part 2. The Lumbar and several Portions of the Sympathetic, and the Nerves of the Abdominal Viscera—Part 3. The Cerebral Nerves—Part 4. The Spinal Nerves. By Joseph Swan. Part 1, two Guineas. Folio. Longman's London, 1830.

☞ *This is a magnificent Work, unequalled by any English plates for accuracy, and unrivalled for boldness and clearness of delineation. Mr. Swan deserves every encouragement, and we trust that the more affluent members of the profession, and the various Medical and Book Societies will extend their patronage to the undertaking. We wish the indefatigable author every success.*

---

5. The Philosophy of Sleep. By Robert Macnish, Author of the Anatomy of Drunkenness, &c. Duodecimo, pp. 268. Glasgow, 1830.

☞ *Mr. Macnish is very favourably known for his Anatomy of Drunkenness, a little work which has received the commendation of very able men. The present volume does no discredit to Mr. M. and perhaps, we may be enabled to make an extract or two from its pages.*

---

6. Elements of Chemistry. By Andrew Fyfe, M.D. F.R.S. E. &c. Second Edition, comprehending all the recent Discoveries. Octavo, pp. 1061. Edinb. Adam Black, 1830.

☞ *It is unnecessary for us to dwell on the merits of this valuable work; they have been stamped by the universal approbation of all who have perused it. To the student in chemistry, and, we may add, in medicine, it will prove an acquisition of the highest value.*

---

7. A Practical Treatise on the Diseases of the Eye. By William Mackenzie, Lecturer on the Eye in the University of Glasgow, &c. Octavo, pp. 861. Longman's, London, 1830.

---

8. The Anatomy of the Human Body, illustrated by 158 Plates, taken partly from Nature, partly from the most celebrated Authors. By Andrew Fyfe, Fellow of the Royal College of Surgeons of Edinburgh. Quarto. Edinburgh, 1830.

Also: Descriptive Letter-press of the same, in 1 vol. 8vo. pp. 233.

☞ *The approbation of the profession has long stamped Fyfe's Anatomy as a valued and valuable work. In the volumes before us, the Plates are separate from the descriptive portion of the work, and are accompanied by a book of Tables, pointing out the parts delineated in the Plates. They are well worth the attention of students. Whether there is likewise a new edition of the Descriptive Anatomy we cannot say, as the able author has not sent it with the rest.*

---

9. A Treatise on Auscultation, illustrated by Cases and Dissections. By Robert Spittal, late Physician's Assistant, and now House-surgeon of the Royal Infirmary, Ed. Octavo, pp. 280. Edinb. and London, November, 1830. *Thirteen Plates.*

---

10. Elements of the Pathology and Practice of Physic. By J. Mackintosh, M.D. &c. Volume the Second, 1830.

☞ *This finishes the Work, which now presents a respectable compendium of practical medicine.*

---

11. Recherches Experimentales sur le Sang, considéré à l'Etat sain, faites pour determiner les Modifications auxquelles est sujette dans l'Economie, la Composition de cette Humeur, et apprecier les Phenomènes physiologiques qui s'y rapportent. Mémoire presentée à l'Institut, Academie des Sciences, en 1828. Par Prosper Sylvain Denis, M.D. &c.

---

12. Elements of Surgery. By Robert Liston, Fellow of the Royal College of Surgeons in London and Edinburgh, &c. Part 1. 8vo. pp. 318. London and Edinburgh, 1831.

---

13. Pathological Observations; Part 3. On Typhoid, Inflammatory, and Symptomatic Fevers; Small-pox after Vaccination, Gout, Rheumatism, Dropsy, Delirium Tremens, and Pemphigus, &c.; with an Appendix of Cases, illustrating the Nature and Treatment of Diseases, more especially in the occasional Substitution of Millefoil and Dandelion for Foxglove, Prussic Acid, Mercury, Colchicum, &c. By WILLIAM STOKER, M.D. &c. Octavo, pp. 138. Dublin, 1830.

---

14. Essays on the Medical Institutions of Naples, No. I. By Dr. ROCHE, of Philadelphia. Octavo, pp. 40.

☞ *A very talented and erudite brochure. When finished, we shall not fail to notice the work.*

---

15. Engravings of the Bones, Arteries, and Nerves, copied from the Works of Scarpa, Soemmering, &c. By EDWARD MITCHELL, Engraver, with explanatory Letter-press, by Dr. KNOX. Second Ed. Edinburgh, 1830.

☞ *We have noticed this highly valuable and meritorious performance in our present Number.*

---

16. Lectures on Anatomy, interspersed with practical Remarks; Vol. II. By BRANSBY B. COOPER, F.R.S. Surgeon of Guy's Hospital, Lecturer on Anatomy, &c. Large 8vo. 2 Plates, pp. 308. Highley, 1830.

☞ *We have noticed the first volume of this valuable Work on a former occasion. We shall advert to the present in the body of this Journal.*

---

17. Bethlem Hospital. Minutes of Evidence taken by the Committee appointed to inquire into the Charges preferred against Dr. WRIGHT, with his Answer. By Dr. WRIGHT. 8vo. sewed, 1830.

☞ *We have given an opinion in another place on this inquiry*

---

18. Illustrations of Mr. Cooper's Surgical Dictionary; published monthly, each Fasciculus containing from four to seven Lithographic Plates, with Letter-press Descriptions, and References to the Text. Octavo, stitched, price Two Shillings each Part; Parts 1, 2, 3. Longman's 1830.

---

19. Science without a Head; or the Royal Society dissected. By One of the 687 F.R.S's. 8vo, pp. 122. Nov. 1830.

☞ *A keen dissection.*

---

20. A new Mode of ventilating Hospitals, Ships, Prisons, &c. being an efficient Method of destroying Contagion, &c. By GEORGE HAWTHORNE, M.D. Octavo, pp. 84. 1830.

---

21. Medicine no Mystery. Second Edition, Nov. 1830. By JOHN MORRISON, M.D.

---

22. Inquiries concerning the Intellectual Powers, and the Investigation of Truth. By JOHN ABERCROMBIE, M.D. Octavo, pp. 435. London and Edinb. December, 1830.

---

23. Cases illustrating the Efficacy of various Medicines administered by Inhalation, in Pulmonary Consumption, in certain Morbid States of the Trachea and Bronchial Tubes, attended with Cough, and in Asthma. By Sir CHARLES SCUDAMORE, M.D. &c. Octavo, pp. 113, Nov. 1830.

---

24. Medical Botany, &c. By JOHN STEPHENSON, M.D. and JAMES MORSS CHURCHILL, F.L.S. &c. Various Numbers.

☞ *For a notice of this useful publication, we refer to the Periscope of the present Number.*

---

25. A System of Operative Surgery, containing a Description of the most approved Plans of performing the different Operations in Surgery on the Dead Body, with Remarks on their Anatomy, and accompanied by practical Observations; being principally designed for the Use of Students in surgery. By W. HARGRAVE, A.M. M.B.C.D Mem. of the Royal College of Surgeons in Ireland, Lecturer on Anatomy, Physiology, and Operative Surgery, &c. Octavo, pp. 530, 1831. Price Nine Shillings.

---

26. Anatomical Demonstrations; or Colossal Illustrations of Human Anatomy. By Professor SEERIG. Translated from the German, Part I. London, published by A. Schloss, Foreign Bookseller, 44, Southampton Buildings, Chancery Lane, 1831. Part 1, containing 4 Plates, price 8s. 6d. each Part, or 12s. colored.

☞ *These are capital Plates, and have our unqualified approbation. Their accuracy is unimpeachable, their execution admirable, and we think that Mr. Schloss is deserving of the highest praise, and, what is more to the purpose, patronage, in entering on such an undertaking. Among the subscribers to this beautiful and useful work, we observe the principal anatomical teachers, Sir Astley Cooper, and Mr. Brodie, names which must carry great weight and influence along with them. On the whole, we cordially recommend the Work to our professional brethren.*

